# Utilization of SDN Technology for Flexible EtherCAT Networks Applications

**DOI:** 10.3390/s22051944

**Published:** 2022-03-02

**Authors:** Ireneusz Smołka, Jacek Stój

**Affiliations:** Department of Distributed Systems and Informatic Devices, Faculty of Automatic Control, Electronics and Computer Science, Silesian University of Technology, 44-100 Gliwice, Poland; jacek.stoj@polsl.pl

**Keywords:** software defined networks, networked control systems, real-time, industrial ethernet, EtherCAT, OpenFlow, OpenWRT

## Abstract

At the beginning of the current century, Ethernet-based communication networks began to be implemented in industrial applications. Some previously used protocols were migrated to Ethernet networks, while many others were strictly developed for this communication medium. Numerous industrial Ethernet protocols do not deliver all the capabilities provided by the Ethernet. For example, limitations may arise associated with wireless communication, use of dedicated switching devices, or operation solely for certain topologies. On the other hand, new technologies are now available, such as software defined networks (SDN), that add new features to Ethernet-based communication systems. In this paper, an EtherCAT network in combination with SDN is analyzed. EtherCAT network may only consist of devices with an implemented EtherCAT protocol stack. Therefore, regular Ethernet switches cannot typically be used in this network and, hence, special network infrastructure may be required to create topologies other than standard line topology. It is shown, however, that this limitation can be overcome by the application of SDN. In addition, a definition of datagram forwarding rules (called SDN flows here) is given, and we demonstrate that EtherCAT datagrams can be sent through routes that are required for proper EtherCAT network operation.

## 1. Introduction

Efficient communication systems for industry are indispensable nowadays. New industrial systems are most often distributed, and they consist of many devices connected by networks. For many years, data exchange between nodes in industrial communication systems was based on fieldbuses. However, in the early 2000s, an Ethernet standard began to be extensively implemented for this purpose. Many protocols used for fieldbuses migrated to, after some adaptations, modern Ethernet communication links, e.g., Modbus TCP [1]. In addition, other protocols were developed strictly for the Ethernet, these include:

- Ethernet Powerlink EPL, introduced by the company B&R (Eggelsberg, Austria) in 2001.
- Ethernet/IP managed by ODVA (Open DeviceNet Vendors Association), launched in 2000.
- Profinet and EtherCAT, both presented for the first time at Hannover Fair in 2003.

Soon afterwards these protocols, alongside some others, became part of the international standard IEC 61158/IEC 61784-2 [2] and, today, they are among the most recognizable in the domain of industrial automation. It is noteworthy that another important protocol, which is not defined in this standard, is known as OPC UA; its first version was released in 2006 [3,4].

Even though numerous protocols were developed solely for application as an Ethernet standard, they cannot take advantage of all the characteristic features of an Ethernet network. The limitations start from the temporal requirements, typical in industrial systems where operation in real-time is necessary. For example, in an EtherCAT network every device is a node in the network with the protocol implemented onto them. As a result, the usage of a common Ethernet infrastructure is not allowed in this case. The aim of this paper is to present a method to overcome this limitation.

EtherCAT communication is based upon the sending of consecutive EtherCAT datagrams from one EtherCAT slave device to another and then returning it to its origin, i.e., the EtherCAT master device [5]. Therefore, the communication is performed in a “logical ring”. Such communications can only be realized with a proper datagram flow—through EtherCAT nodes in a specific order. As a result, the usage of regular Ethernet switches is not possible because it would lead to an incorrect forwarding of the EtherCAT datagrams. A solution is to use software defined networks (SDN), which represents yet another type of virtualization that is already employed in computer systems [6].

In SDN, the forwarding of datagrams by switching devices accords to the programmed flows. Such switches allow the routing of the datagrams along specified paths; thus, EtherCAT communication through non-EtherCAT infrastructure becomes a possibility. This solution, called here as EtherCAT over SDN (EOS), is presented in this paper.

The latter is organized as follows. The following section presents previous works that relates communications in industrial systems to protocols based on the Ethernet, such as the EtherCAT. Section 3 describes the concept of SDN in application to EtherCAT-based systems. In Section 4, the experimental testbed is presented and the results from which are given in Section 5. Section 6 includes a discussion on these experimental results. The final section presents the conclusions based on our solution and offers future research possibilities.

## 2. Related Works

The operation of industrial computer systems is concurrent with production plant and factory floor processes. Therefore, computer systems must respond to events that occur in the processes in real-time, i.e., in a timely manner [7]. The early version of the Ethernet standard was difficult to introduce to industrial real-time systems. It was caused by the CSMA/CD media access mechanism which made delays in data transfer unpredictable [8]. In this so called “shared Ethernet” technique, real-time communication had to be implemented at the application level. An example of such a protocol is Ethernet PowerLink EPL, in which the producer–distributor–consumer mechanism is implemented [9]. Here, communication is coordinated by a predefined manager called the Managing Node MN (i.e., the distributor of the data), which decides when other network nodes can transmit (or produce) their data. When the transmitted data are broadcast, they may be read (consumed) from the network by the MN, as well as the other nodes since most of the network devices are both producers and consumers of data. Therefore, in EPL networks, each device is indistinguishable, and they are called Controlled Nodes (CNs). At any given point in time, only one datagram is transmitted in the network—either by the MN or one of the CNs. Therefore, the data flow is clearly describable in terms of time. In other words, the time needed to distribute a dataset in the network is easily calculated [10].

The later development of a “switched Ethernet”, and a full-duplex communication system, allowed for an easier introduction of Ethernet networks to industrial systems, since new protocols capable of real-time operation had been created. One of the many examples is Ethernet/IP, managed by ODVA, which combines the Ethernet standard and TCP/IP communication with CIP (Common Industrial Protocol) at the application level [11]. Here, data exchange is based on the provider-consumer principle. Yet, another example is the Profinet protocol [12], in which the communication model consists of three types of devices: an IO-Controller that coordinates communication, IO-Devices that exchange input–output (IO) data with the IO-Controller, and a IO-Supervisor that is required for network configuration and diagnostics (however, this is not needed during the normal operation of the network) [13]. The three aforementioned protocols are among the most popular for use in industrial applications. Similar to the EtherCAT protocol that is described in the next section.

New protocols continued to improve the applicability of the Ethernet standard to industrial applications. Alongside which the spatiotemporal description of the network operation and data flow has gained complexity. From this point of view, for a given industrial communication network that is known as the networked control system (NCS), the latency of data exchange should be calculated and analyzed. Previous research is available that compares the latency of aforementioned Ethernet protocols, such as Profinet and EtherCAT networks [14]. However, more recent works instead focused on the quality of the time synchronization. In an EtherCAT network, the latter is realized by use of Distributed Clock mechanisms, which can be improved by using system time drift compensation [15]. A solution, similar to those based on a Distributed Clock, has also been implemented on a Linux platform [16], which involved a time synchronization setup between Linux and the EtherCAT master [17].

Another area of research concerns industrial control systems (ICS) and the detection of anomalies in EtherCAT networks. Using machine learning techniques, the network traffic can be classified and various attack types flagged [18]. Anomaly detection may be also based upon device-level periodicity that can detect denial of service (DoS) and code-injection attacks [19]. Outside of cybersecurity, reliability is especially crucial in production systems. Ref. [20] describes a reliability enhancement method based on game theoretic feedback control. Of course, in order to improve the reliability of EtherCAT networks, a medium redundancy may also be applied by using a ring topology [21]. Other papers also mainly focus on EtherCAT applications; for example, in connection to the robot control of the EtherCAT master [22,23] or the design of the EtherCAT slaves [24].

In many protocols except EtherCAT (for example, Profinet), the communication network can be extensively similar to a typical Ethernet network. In such cases, common switches and wireless communication may be applied [25]. Moreover, for EPL and Ethernet/IP, there is no special hardware requirements and various topologies can be created. This type of flexibility is unavailable in EtherCAT networks because every component of such a network is required to be an EtherCAT network node and, thus, common switching devices cannot be used. Wireless communication is also unfeasible, except for higher level communication that use an EtherCAT Automation Protocol (EAP). Although an EtherCAT wireless extension [26] is available, which refers to acyclic communication that employs a Type 12 process data unit (PDU) and mailbox frames, it cannot be used in I/O data transfer.

The concepts presented in this paper could overcome these limitations by using SDN. It is shown that realization of EtherCAT communication using non-EtherCAT switches is obtainable. It means that, instead of using only line topology (which is typical for an EtherCAT network) or employing dedicated EtherCAT junction modules (for creation of topologies other than line ones), it is possible to create any topology using SDN switches. Thus, it makes the network structure more flexible and enables the possible ability to dynamically change the topology in order to connect new EtherCAT devices. Using SDN also creates another advantage, in that the sharing of the same network infrastructure for both EtherCAT and regular IT communication.

## 3. EtherCAT over SDN

In EtherCAT networks there are two types of communication methods: the EtherCAT Device Protocol (EDP) and the EtherCAT Automation Protocol (EAP). These are described below. The concept of software defined networks is then presented, followed by the description of the EtherCAT over SDN solution.

### 3.1. EtherCAT Protocol

Communication using EtherCAT Device Protocol, which is typically simply called the EtherCAT protocol, is based upon a master–slave principle. However, in contrast to other master–slave communication networks (for example, the well-known Modbus communication network or Profibus, in which the master–slave principle is applied alongside the token passing mechanism between the master stations), the EtherCAT master station does not send separate datagrams that exchanges data with different slave stations using the request–response approach. EtherCAT datagrams contain processing data that propagates through all the EtherCAT slaves, which are called EtherCAT Slave Controllers ESC. This means that only one datagram is required for communicating with many ESC, which is then processed by the latter without the need for data buffering and storing. Therefore, minimal latency for the propagation of the datagram is introduced [20] and the exchange process for the input and output data becomes highly effective. In detail, when a datagram arrives at an input Ethernet port of a given ESC it is efficiently forwarded almost concurrently to its output port. The output dataset is extracted from the datagram “on-the-fly”; the input state is written similarly to the datagram.

The processing of the datagram is implemented by computer hardware in the form of a Fieldbus Memory Management Unit (FMMU) of the ESC. Datagrams traverse through FMMUs with a latency as low as 1 µs for slave stations equipped with at least one Ethernet interface (100BaseTX or 100BaseFX) and 300 ns for slaves with only low voltage differential signal (LVDS) ports (this is also called an E-Bus interface, in devices by Beckhoff (Verl, Germany), which is used as a backplane connection in terminal modules such as input and output modules [27].) EtherCAT datagrams are transferred by just using Ethernet frames with broadcast MAC addresses. In other words, in reference to the ISO/OSI model, only the physical and data link layers (defined in Ethernet standard) are implemented; protocols from the upper layers are not used. In particular, no IP addressing is required since EtherCAT is only concerned with the application layers.

Apart from EDP, which is briefly described above, there is also the possibility of using EAP. This protocol involves communication between the master stations and it is based on the producer–consumer principle. However, the details on EAP are not described in this paper since we solely focus on EDP (for simplicity, this is just called EtherCAT later); the presented solution can, however, be applied to both. In EAP, “typical” Ethernet network devices and “typical” datagram processing can be used (but “on-the-fly” processing does not apply).

**Figure 1:**
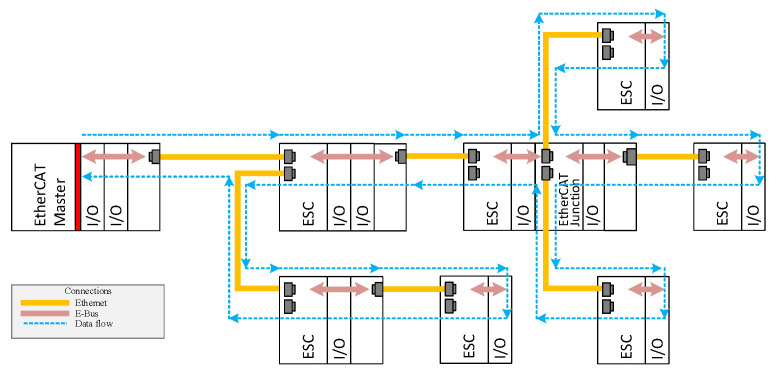
Schematic showing the EtherCAT network, the topology for which is always a logical ring.

EtherCAT Device Protocol operates in a logical ring. Every datagram traverses from one ESC to another in a consecutive fashion. By taking advantage of full-duplex communication, the final topological ESC returns the EtherCAT datagrams to the master station, as shown in Figure 1. As stated earlier, it is not possible to include regular Ethernet switches in an EtherCAT network; one of the reasons is the need for broadcast MAC addressing. When a tree or star topology is built, a module called an EtherCAT junction should be used (also displayed in Figure 1).

For the above reasons, common IT infrastructure cannot typically be utilized in EtherCAT communication, although its application could be made possible by using the Time Sensitive Network concept [28]. Since 2021, an IEEE task group has been attempting to standardize the real-time and safety of networks [29], and apply a critical enhancement for the Ethernet [30]. Unfortunately, we authors have not found any information on the practical application of TSN in industrial scenarios. Hence, we present another solution based on SDN.

### 3.2. Software Defined Networks

In traditional Ethernet networks, the decisions on the routing of the datagrams are made by the switching devices. In SDN, an additional autonomous device is used that controls the data traffic—this is called the SDN controller. In SDNs, the control plane is separated from the data plane and the central SDN controller coordinates the data flow within the network. SDN only switches forward the datagrams according to the SDN controller instructions, which is known as the flows [31]. Great advantages may arise by decoupling the control and the data plane alongside a control of the centralized network. Above all, in such a scenario, decisions made by the SDN controller are based on a global view of the network. Therefore, all the decisions are more relevant to the current condition of the whole network [32].

SDN may be applied in various areas: Internet of Things [33] and Wireless Sensor Networks [34,35,36], industrial systems [37], or on the factory floor [38]. They can be implemented to improve the management and maintenance of networks [39], as well as increasing the security by use of Intrusion Detection Systems [26], protecting against eavesdropping attacks [40,41], creating resilient networks [42], and enhancing network reliability [43]; related papers also concern communication determinism [27]. In further reference to SDN, the Future Industrial Network Architecture (FIND) defines a guidance on the industry-related requirements in communication systems development [28]. For our purposes, SDN is applied so that datagrams forward to different ports of the SDN switching devices (called OVS or vSwitches). Moreover, every datagram is forwarded to the hosts of the networks, consecutively, in a predefined order. As a result, the topology resembles a line topology that corresponds to datagrams routing within EtherCAT networks.

### 3.3. EtherCAT over SDN

As stated earlier, a regular Ethernet infrastructure cannot be used within EtherCAT networks because a significant part of the datagrams in the EtherCAT is sent as broadcasts. Moreover, the broadcasted datagrams must reach every slave device in consecutive order. While typical Ethernet switches cannot cope with this type of datagram forwarding, the OVS within SDN are capable of this task. Despite the various addressing possibilities for the datagrams (i.e., either broadcasted, multicasted, or unicasted), forwarding by OVS is possible for any required scenario in a consecutive manner that accords to the needs of every slave device. By defining the appropriate flows in the SDN network, datagrams routing can be freely chosen according to, for example, the type, length, or payload of the datagrams.

## 4. Experimental Research

**Figure 2:**
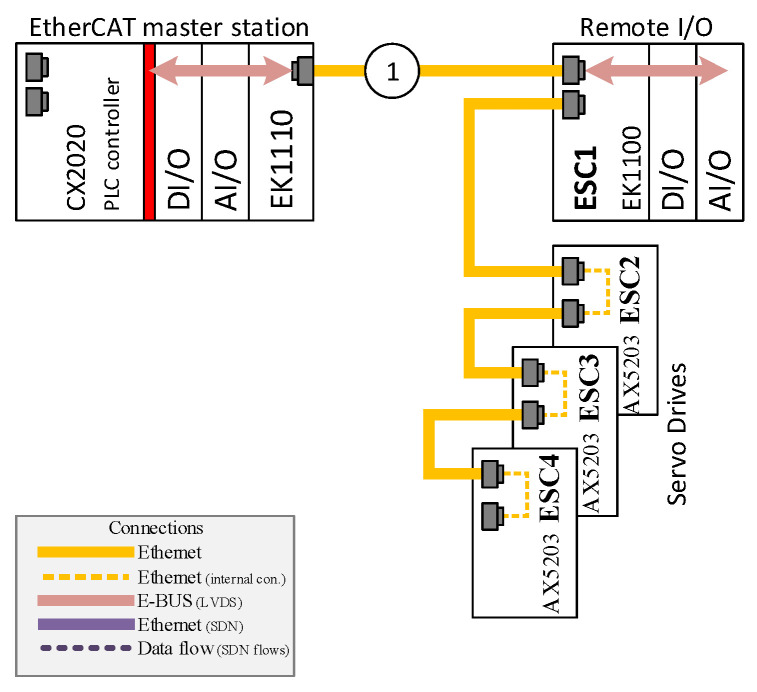
Schematic of the EtherCAT network, in a typical line topology configuration, used during the experimental research. The encircled digit “1” denotes the ET2000 probe location (measurements point no. 1).

**Figure 3:**
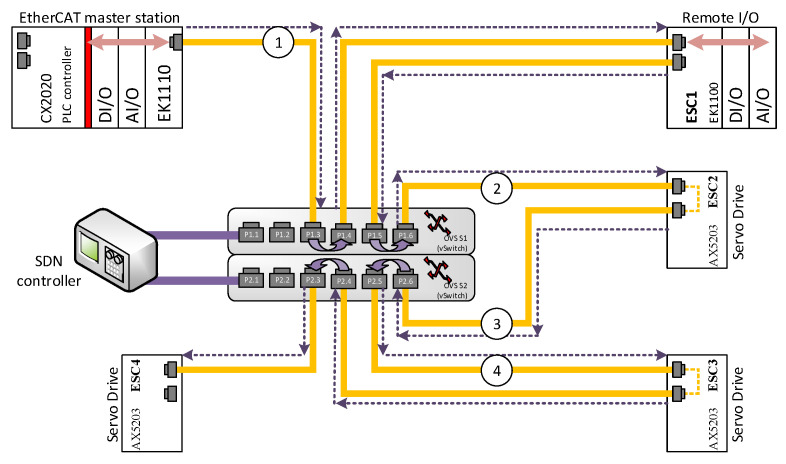
Depiction of the EtherCAT over SDN (EOS) network solution in a star topology. The encircled digits “1” to “4” denote the ET2000 probe locations (measurements points no. 1 to 4).

The concept of EtherCAT over SDN (also known as EOS) was verified during the experimental research. The testbed consisted of one Beckhoff controller (a EtherCAT master) and four ESC devices: EK1100 with some I/O modules. The EtherCAT network components were connected in two ways:

- A regular EtherCAT network in a line topology, which uses the switches that are integrated into the devices (see Figure 2).
- An implementation of the EOS concept, so that the ESC is connected to the master station via OVS (see Figure 3).

In Figure 2, a typical EtherCAT network is presented that contains one master station and four ESC devices connected in a line topology. This is the most common topology in EtherCAT networks, since every ESC device contains an Ethernet switch built into the network interface. Other topologies are also possible, but they require the use of additional modules such as the EtherCAT extension modules (e.g., EK1110 by Beckhoff) or the EtherCAT junction modules (e.g., EK1122 by Beckhoff) that are mentioned in the previous section (see Figure 1). An identical set of EtherCAT devices, which are connected to an SDN network, representing the EtherCAT over SDN (EOS) solution is shown in Figure 3. For this scenario, any physical topology is possible; here, a star topology was chosen (as depicted in the figure) in order to present a topology that is significantly changed from the original line topology.

Using the EOS solution, a logical line topology that is typical for EtherCAT networks is realized by using an appropriate definition for the SDN flows. According to such flows, every datagram that is sent by the EtherCAT master is received on port P1.3 of the S1 OVS and then forwarded to port P1.4, which is where the ESC1 (remote I/O station) is connected. Every datagram that derives from the ESC1 slave on port P1.5 is forwarded to port P1.6, which is connected to the ESC2 slave, and so on. The datagrams eventually reach ESC4 on port P2.3, from which the ESC4 datagrams return to the master station via the same route (the opposite direction required for this is not marked on Figure 3). Of course, while the datagrams travel through every ESC device, the IO data associated with the given device are processed, i.e., the output and input data are read from and written to the datagrams, respectively. This type of data processing is based on a “on-the-fly” approach, which means that the IO data can be processed even before the whole datagram is received from the network. The presented concept was verified during the experimental research; this is described in the next section. In greater depth, the main goal of the experimental research was to verify if EtherCAT communication can be coordinated in a SDN network, which was shown to be possible.

It is also important to determine the temporal costs that the OVS introduce to the EtherCAT communication network. Measurements on the network traffic were performed to obtain this necessity. For this purpose, an ET2000 multi-channel probe by Beckhoff was used. This enabled the capture of Ethernet datagrams with precise timestamping at the four monitoring points of the network; these are marked by encircled digits from “1” to “4” in Figure 3 reffered latter as measurment points. The datagrams were registered using a regular PC with Wireshark software. Analysis of the timestamps enabled the measure of the network latencies, which are introduced by the devices located between the monitoring points. The timestamps are added, by the ET2000 probe, at the end of every Ethernet datagram. Therefore, the Ethernet traffic analysis is not dependent on the device used for capturing the datagrams. The timestamps had the resolution of 1 ns; however, the actual accuracy of the timestamping according to the vendor is 40 ns [44], whereas the delay in communication that the probe introduces to the network is lower than 1 µs.

For the testbed illustrated in Figure 3, the following components were used:

- EtherCAT master: CX1020 embedded PC by Beckhoff;
- ESC: remote I/O station based on EK1100 EtherCAT ECS module with four EtherCAT I/O modules;
- SDN switches (OVS): virtual switch implementation based on Mikrotik RB2011UIAS-2HnD-IN;
- SDN controller: OpenDayLight controller installed on a PC with the Ubuntu operating system.

The two switches by Mikrotik (Riga, Litvia) that were used had their firmware changed to the OpenWRT and OpenvSwitch package. In addition, the L2 functionality of the switches was disabled. Therefore, without the defined SDN flows, the switches cannot provide any forwarding for the datagrams. Configuring the SDN flows required OpenFlow Manager, which was used to define the flows as described above. These flows did not contain any filtering, so that every datagram received on port P1.3 was forwarded to port P1.4, and so on.

## 5. Results

**Figure 4:**
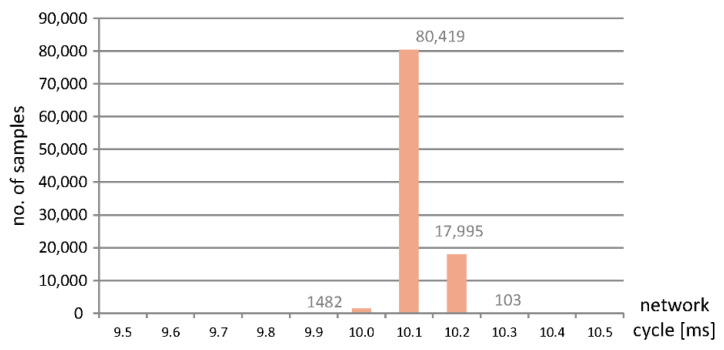
Histogram showing the update time of the EtherCAT ESC in a network without the SDN.

**Figure 5:**
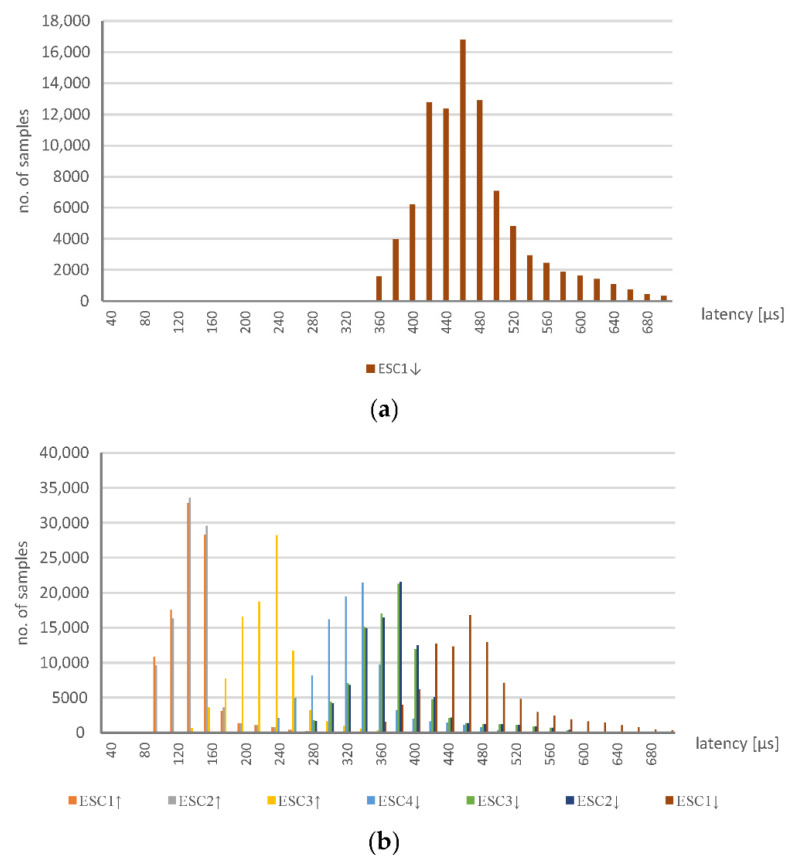
Histograms for the EtherCAT with SDN: (**a**) measurements taken at point no. 1, which provide the latency time for datagram routing through all the devices, and (**b**) measurements at points no. 1 through 4.

The Ethernet network traffic was captured for the two system topologies, i.e., with and without application of the EOS solution, as depicted by Figure 2 and Figure 3. The results of which are presented in terms of the histograms of Figure 4 (for a system without SDN) and Figure 5 (with SDN and the EOS solution applied). In the experiment, 100,000 datagrams were captured and analyzed. In the former case, without the SDN, the *x*-axis represents time in the units of microseconds, while the *y*-axis is the number of measurements at a given network cycle, which was configured to be 10 ms. Most of the results reside at 10.1 ms, although signficant numbers are seen at a slightly longer time. In general, the communcation data remain within the configured cycle time with a 2% jitter. This shows that the latencies introduced by the ESC are minimal; the specifications of which are described in Section 3. Datagrams traverse through the FMMUs of the ESCs with latencies as low as 1 µs for the slaves that contain at least one Ethernet interface (100BaseTX or 100BaseFX) and 300 ns for the slaves with only LVDS ports. The delay introduced by the ESC2-ESC4 and the module EK1100 of ESC1 equates to 1 µs. While every I/O module of the ESC1 creates a latency of 300 ns. A total latency as little as 1.2 µs, for the four modules, is thus highly plausible.

The system with the implemented EOS solution was more thoroughly tested. Measurements at the four different points were taken, which enbled a calculation of the EtherCAT datagrams flow in terms of latency time.

Figure 5a represents the variation in latency for the EtherCAT datagrams with the EOS solution implemented. Every measurement corresponds to the latency of a datagram that is transferred through the network. For example, over 16,000 datagrams each required a latency time of 460 µs to be transferred from the PLC to the final ESC device and returned. Here, in contrast to Figure 4, only the latencies are presented with no reference to the network cycle time (which was again set at 10 ms). For the EOS solution, compared to the system without SDN, the latency values are significantly greater and the distribution of them is much more spread out. The most likely measurement is 460 µs and over 90% of the measurements reside in a range from 360 to 680 µs—the jitter is, thus, much greater when the EOS solution is applied. This dataset is represented in the figure by ESC1^RX^, which refers to the time period that begins when the datagrams are sent from the PLC (through the measurement point no. 1) and ends when the returned datagram is received from ESC1. In other words, the time required for the datagram to travel through the ESC1, ESC2, ESC3, and ESC4 devices and back. In an EtherCAT network, this involves one datagram that is consecutively processed by all the ESC.

The latency of ESC1^RX^ is calculated based on the datagrams captured with the ET2000 device at the measurement point no. 1. For every measurement point, the two Ethernet interfaces with the ET2000 probe are related. Datagrams that are received on one port are then forwarded to another port (which applies to every measurement port) and every pair of ports creates a monitoring channel. Additionally, the incoming datagrams are duplicated and sent to the monitoring port (in detail, the uplink—nineth Ethernet port of the ET2000 probe is used, while the ports of the monitoring points operate in the 100 Mbs mode and the uplink, i.e., the port with the duplicated datagrams, is a 1 Gbs port). The monitoring port is connected to the capturing device—namely, a PC with Wireshark software. The duplicated datagrams are extended by a status field (called the EtherCAT switch link), which consists of the port number that corresponds to when the datagram was received and a timestamp. For the measurement point no. 1, the calculations for the delay between receiving the datagrams from the PLC and the returned datagrams from ESC1 are determined. Other descriptions, similar to ESC1^RX^, are displayed in Figure 5b. They refer to the following measurements:

- ESC1^TX^—the latency between the datagrams received from the PLC at measurement points no. 1 and no. 2. It determines the time required for the datagrams to travel from point no. 1 to 2.
- ESC2^TX^—the time needed for datagram transfer from point no. 1 to 3.
- ESC3^TX^—as above, but between points no. 1 and no. 4.
- ESC4^RX^—the latency between the datagrams received from the PLC at point no. 1 and the returned datagrams arriving at point no. 4. It determines the time need for datagram transfer from point no. 1 to point no. 4, which is added to the time taken from ESC4 back to point no. 4.
- ESC3^RX^—as above but applies to datagram transfer through points from no. 1 to point no. 4, then ESC4 back to point no. 3.
- ESC2^RX^—as above but with back transfer to point no. 2.
- ESC1^RX^—the latency for the transfer of datagrams from point no. 1 to ESC4 and the return journey to point no. 1.

A summary of the mean and median of the measured results is presented in Table 1. The difference between the ESC1^TX^ and ESC2^TX^ values is very small, because it refers to the latency introduced by the ESC2 device only. According to its specifications it should be 1 µs, which is consistent with the presented results. The variation between ESC2^TX^ and ESC3^TX^ corresponds to the latency of the OVS—which is around 85 µs. However, the difference between ESC3^TX^ and ESC4^RX^ includes two passages through the OVS switches and one through the ESC4; here, the difference is around 100 µs. Moreover, the value of ESC1^TX^ includes a double routing through the OVS S1 switch, which shows some discrepancy since it has an average value around 147 µs. However, this inconsistency may result from not using a very powerful device, which is made from a regular OpenWRT switch, in the role of the OVS. It is expected that the results will be much more consistent when a dedicated OVS is used. Nevertheless, the experiment shows that the EOS solution can operate as required, although its temporal characteristics should be fully considered when applied to real-time systems. It is noteworthy that a similar OVS switch could be implemented on a Raspberry Pi device, which is equipped with Ethernet interfaces connected to the USB ports. However, the latency introduced by this type of switch could be much more significant [45].

## 6. Discussion

Our experimental research demonstrated that, by the implementation of SDN technology, it is possible for an appropriate routing of EtherCAT datagrams to create communication in EtherCAT networks via SDN. The definition of flows for the EtherCAT datagrams using OVS enable the ESC devices to operate seemingly in a line topology, while the actual (physical) architecture may not have this topological characteristic at all. As a result, the EtherCAT traffic can be routed through regular IT infrastructure, which enables efficient real-time interconnects between different areas of a production plant or factory floors without a need for elaborate and expensive devices.

Importantly, inclusion of additional devices in the EtherCAT network (i.e., the vSwitches/OVS) will introduce some extra latency to the datagram transfer. In the testbed experiment used, this was measured to be around 85 µs. On a comparison of this value with the latency of the EtherCAT slaves, which are as low as 1 µs or even 300 ns (depending on the type of slave device used), the OVS latency may seem to be overly large. Nevertheless, in most industrial applications, the network cycle is expressed in milliseconds rather than microseconds. Therefore, even with these types of delays, the presented solution can be effectively applied to real scenarios. Nevertheless, of course, it cannot be employed when high-precise communication is required, for example, in the synchronous operation of servo drives; in most cases, however, it should be applicable. In addition, the measured latency applies to the general-purpose switches (the Mikrotik) that were used in the testbed experiments, where the firmware is changed to OpenWRT. It is expected, therefore, that more advanced solutions that significantly minimize the latency in such cases should be possible.

Another drawback of the presented solution is that double Ethernet cables are necessary for the connection of every ESC to the OVS. Importantly, these additional cables are only required between an OVS and the first ESC, since the other ESC can be connected directly to the first ESC in a line topology. In future work, there are plans to eliminate this requirement, so that the operation of the OVS is similar to the EtherCAT junction module shown in Figure 1, in which only one Ethernet cable for every ESC is needed.

## 7. Conclusions and Future Work

In this study, we demonstrated that SDN technology can be implemented in the region of industrial Ethernet applications and it may overcome some of the limitations associated with EtherCAT networks. Typically, for example, EtherCAT networks are required to have a line topology when no EtherCAT junction modules are employed and regular Ethernet switches cannot usually be applied. We have demonstrated that both of these limitations can be removed by the use of SDN. Application of common switches, such as OVS (or SND switches), may deliver flexible network infrastructure even when industrial communication protocols such as EtherCAT are used. Moreover, common IT network infrastructure become useable in such networks if it supports SDN. Another advantage is that changes to the structure of the network is possible by a modification of the program, so that physical rewiring of the network can be avoided. To enable system architecture to be even more flexible, future works plan to implement wireless communication in EtherCAT networks, since it should be possible to define SDN flows that enable the routing of EtherCAT datagrams in a Wi-Fi network. The general intention, for this future research, is to interconnect more than two devices with one wireless network.

The presented solution is applicable not only to EtherCAT networks but also systems based on other protocols. For example, in EPL networks, only one distributor node may be used, called the Managing Node (MN), which creates one real-time domain. When additional MNs are present, separate Ethernet infrastructure should, in general, be used. Moreover, different EPL real-time subsystems may coexist on the same infrastructure, which has applications to managed Ethernet switches and the logical separation of the domains by a VLANs configuration. However, such domain separation could be based on SDN in future. It would increase the security of the EPL by not forwarding datagrams that arrive from other ports of the OVS (SDN vSwitches), which is where the controlled nodes (CN) are connected; for example, those from outside the processing network.

## Figures and Tables

**Table 1:** A summary of the latency measurements (in units of µs) in terms of the average (the mean) and the median values.

	SDN (EOS Solution Implemented)						
	ESC1^TX^	ESC2^TX^	ESC3^TX^	ESC4^RX^	ESC3^RX^	ESC2^RX^	ESC1^RX^
AVG	147.3	148.7	234.1	334.7	380.7	381.9	474.7
MEDIAN	133.9	135.1	222.0	319.3	363.9	365.1	455.6
